# Toward personalized circuit-based closed-loop brain-interventions in psychiatry: using symptom provocation to extract EEG-markers of brain circuit activity

**DOI:** 10.3389/fncir.2023.1208930

**Published:** 2023-08-21

**Authors:** Brigitte Zrenner, Christoph Zrenner, Nicholas Balderston, Daniel M. Blumberger, Stefan Kloiber, Judith M. Laposa, Reza Tadayonnejad, Alisson Paulino Trevizol, Gwyneth Zai, Jamie D. Feusner

**Affiliations:** ^1^Campbell Family Mental Health Research Institute, Centre for Addiction and Mental Health, Toronto, ON, Canada; ^2^Department of Psychiatry, University of Toronto, Toronto, ON, Canada; ^3^Temerty Centre for Therapeutic Brain Intervention, Centre for Addiction and Mental Health, Toronto, ON, Canada; ^4^University Psychiatry Hospital, University of Tübingen, Tübingen, Germany; ^5^Institute for Biomedical Engineering, University of Toronto, Toronto, ON, Canada; ^6^University Neurology Hospital, University of Tübingen, Tübingen, Germany; ^7^Center for Neuromodulation in Depression and Stress (CNDS), Department of Psychiatry, Perelman School of Medicine, University of Pennsylvania, Philadelphia, PA, United States; ^8^TMS Clinical and Research Service, Neuromodulation Division, Semel Institute for Neuroscience and Human Behavior, University of California, Los Angeles, Los Angeles, CA, United States; ^9^Department of Psychiatry and Biobehavioral Sciences, University of California, Los Angeles, Los Angeles, CA, United States; ^10^Division of the Humanities and Social Sciences, California Institute of Technology, Pasadena, CA, United States; ^11^Department of Women’s and Children’s Health, Karolinska Institutet, Stockholm, Sweden

**Keywords:** EEG, TMS, symptom provocation, OCD, anxiety

## Abstract

Symptom provocation is a well-established component of psychiatric research and therapy. It is hypothesized that specific activation of those brain circuits involved in the symptomatic expression of a brain pathology makes the relevant neural substrate accessible as a target for therapeutic interventions. For example, in the treatment of obsessive-compulsive disorder (OCD), symptom provocation is an important part of psychotherapy and is also performed prior to therapeutic brain stimulation with transcranial magnetic stimulation (TMS). Here, we discuss the potential of symptom provocation to isolate neurophysiological biomarkers reflecting the fluctuating activity of relevant brain networks with the goal of subsequently using these markers as targets to guide therapy. We put forward a general experimental framework based on the rapid switching between psychiatric symptom states. This enable neurophysiological measures to be derived from EEG and/or TMS-evoked EEG measures of brain activity during both states. By subtracting the data recorded during the baseline state from that recorded during the provoked state, the resulting contrast would ideally isolate the specific neural circuits differentially activated during the expression of symptoms. A similar approach enables the design of effective classifiers of brain activity from EEG data in Brain-Computer Interfaces (BCI). To obtain reliable contrast data, psychiatric state switching needs to be achieved multiple times during a continuous recording so that slow changes of brain activity affect both conditions equally. This is achieved easily for conditions that can be controlled intentionally, such as motor imagery, attention, or memory retention. With regard to psychiatric symptoms, an increase can often be provoked effectively relatively easily, however, it can be difficult to reliably and rapidly return to a baseline state. Here, we review different approaches to return from a provoked state to a baseline state and how these may be applied to different symptoms occurring in different psychiatric disorders.

## 1. Introduction and background

A promising perspective of therapeutic brain stimulation is that it enables the possibility of circuit-based therapies. Unlike pharmacotherapy (where only the dose can be varied), there are a number of configurable parameters in the application of a brain intervention such as transcranial magnetic stimulation (TMS). For example, in addition to intensity, the stimulation can be targeted to specific cortical areas and be applied with specific temporal patterns. Additionally, the synchronization of the timing of stimuli with individual brain oscillations simultaneously recorded in the real-time electroencephalogram (EEG) provides a new avenue for personalizing this therapeutic approach in order to achieve a specific desired therapeutic change in a dysfunctional brain network. However, the vast parameter space of where and how therapeutic TMS is most effective has barely been explored. It is not feasible to perform a ‘grid search,’ and searching for a protocol that is effective “on average” may not be fruitful since the optimal protocol is likely to vary between patients.

Instead, recent efforts in the neurophysiological domain had been focusing on optimizing personalized therapeutic brain stimulation using concurrent neurophysiological read-outs in the form of concurrent EEG and TMS-evoked EEG (Parmigiani et al., 2023). The motivation is to assess whether the neuroplastic changes induced by TMS are therapeutically effective and optimize the parameters iteratively. Such a TMS-EEG or EEG-derived biomarker that measures the “state” of the circuit on a timescale of minutes is required to implement personalized circuit-based closed-loop brain interventions. However, deriving the state of a specific brain circuit from a few tens of seconds of an EEG signal is not only challenging due to the presence of noise in the EEG (such as ocular and muscle artifacts) but also due to a “curse of dimensionality” (Altman and Krzywinski, 2018) that makes the identification of a reliable mapping from a segment of TMS-EEG data (a matrix consisting of time by channel) to the activity of the circuit of interest at that time (a scalar) difficult.

Reliable EEG state markers of specific neurophysiological processes exist, such as sleep spindles and beta bursts. Different features that can be extracted from the EEG signal with a temporal resolution of seconds (spectral amplitude), fractions of seconds (EEG microstates, coherency-based connectivity states), and even milliseconds (phase of oscillations) are known to reflect underlying neurophysiological processes and have shown promise for personalized therapeutic interventions (Zrenner et al., 2018, 2020; Gordon et al., 2021, 2022). EEG and TMS-EEG signals are also different in wakefulness and sleep and during different pharmacological interventions (Massimini et al., 2005; Sarasso et al., 2014; Ziemann et al., 2015). However, for informing therapy in psychiatric disorders, EEG and TMS-EEG-derived biomarkers from group-level differences between patients and healthy controls (such as frontal alpha power asymmetry) have not translated to a useful method at the individual level. Further, it is unclear whether this is an optimal strategy given the heterogeneity in psychiatric disorders at both the symptom and diagnosis levels.

Whether individual EEG markers of psychiatric symptom severity can be derived using a personalized calibration approach and whether these are more reliable and effective than EEG markers derived from group averages, remains to be tested. We hypothesize that the algorithmic methods used in brain computer interfaces (BCI), where EEG is increasingly effective at decoding brain states with a limited temporal resolution of seconds (e.g., those related to a specific behavior or set of behaviors or circumscribed cognitive phenomena), may also be effective at estimating symptom-related brain states (more broadly involving several, or combinations of, behavioral and cognitive processes) in psychiatric disorders.

**FIGURE 1:**
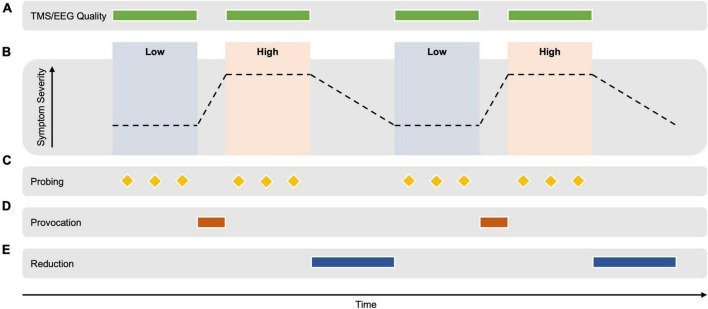
Hypothetical example of an experimental design for extracting TMS-EEG-derived biomarkers of brain circuit activity correlating with the expression of psychopathology. **(A)** TMS-EEG signal quality is monitored and optimized during the measurement. **(B)** TMS-EEG/EEG data is acquired during alternating periods of high vs. low symptom severity. TMS-EEG signatures that can distinguish between the two states are computed using the contrast between the TMS-EEG data of both conditions. The emotional/cognitive state should differ only for the degree of symptom severity but otherwise be identical. Psychiatric state switching needs to be achieved multiple times during a continuous recording to distinguish slow changes in the EEG signal that occur with time (e.g., vigilance, presence of artifacts) from differences in the EEG signal due to the emotional/cognitive state. **(C)** Symptom severity is repeatedly assessed during the conditions using physiological markers (e.g., blink reflex, skin conduction, and heart rate) and the participant’s report (e.g., visual analog scale). **(D)** A personalized symptom provocation intervention shifts the emotional/cognitive state from a “low” to a “high” symptom severity, e.g., as could occur with the threat of shock paradigm (Grillon et al., 1991; Roemer and Borkovec, 1994; Ameli et al., 2001; Åsli et al., 2009; Balderston et al., 2017a,b; Hur et al., 2020), or with script driven imagery in those with PTSD (Lang, 1979; Liotti et al., 2002) and visual images and/or words in those with OCD (Dehghan et al., 2021) and those with specific phobia (Schienle et al., 2007). The specific provocation method is developed prior to the measurement in a calibration session. **(E)** Similarly, a personalized symptom reduction intervention could potentially return the state to a comparatively “low” symptom severity [e.g., personalized focused distraction (Wegner et al., 1987) or a distracting task (Amir et al., 1997) as has been used in experiments in participants with OCD; or with immediate removal of an aversive stimuli, as demonstrated in participants with OCD (Lutz et al., 2008)]. This transition is expected to take longer than the provocation transition. The measurement should occur in controlled laboratory conditions for less than 90 min, repeated on two separate days to assess the reliability of the derived markers. Note that by TMS-EEG, we mean not only the TMS-evoked EEG potentials, but also TMS-induced oscillations, as well as the ongoing EEG signal between the TMS pulses.

Brain computer interfaces methods for the extraction of EEG markers rely on the contrast between cognitive states (e.g., motor imagery), whereby optimized EEG montages (spatial filters) are extracted that optimally differentiate between the two conditions using the difference in the statistical relationships between signals from different brain areas. One such statistical relationship is the covariance matrix, that quantifies the relatedness of the signals measured between every pair of EEG sensors. The resulting contrast in the covariance matrices corresponding to the two conditions can be exploited using the mathematical tool of eigendecomposition in a class of methods termed common spatial patterns (Grosse-Wentrup and Buss, 2008). A combined spatial and temporal decoding approach has also shown promise in the motor system (Metsomaa et al., 2021). By analyzing the difference between the EEG signal extracted during two different cognitive states, the predictive properties of the signal can be determined, while those that are not can be attenuated. In order to avoid a confounding effect of temporal differences (such as change in vigilance and recording properties), it is necessary to switch between the two conditions multiple times to average out slow non-specific changes (see Figure 1). Note that between the switching steps, the conditions should differ only in the degree of symptom expression, with an otherwise similar cognitive state.

Translating this approach to psychiatry requires EEG data during different levels of symptom severity. Some success has been achieved using long-term recordings of brain data while tracking natural fluctuations in symptoms over several days (Etkin, 2018). To derive EEG markers from a recording under laboratory conditions, it is necessary to switch psychiatric symptoms “on and off” repeatedly for a total of tens of minutes during the recording. Effective approaches for a rapidly controlled provocation of symptoms in different psychiatric disorders have been proposed to “switch on” or “activate” the relevant brain circuits before treatment (e.g., in exposure therapy for phobias or before TMS therapy for obsessive-compulsive disorder OCD) (Carmi et al., 2019).

Symptom provocation can be achieved under controlled conditions to evoke a psychiatric symptom or a neurobiological response via psychopharmacological and/or behavioral stimuli, such as contamination stimuli for OCD and videotapes of combat for patients with posttraumatic stress disorder (PTSD) (D’Souza et al., 1999). Symptom provocation has been applied in the study of most psychiatric disorders, including schizophrenia, major depressive disorder, bipolar disorder, panic disorder, OCD, phobias, PTSD, and substance use disorder (D’Souza et al., 1999).

The ability to “switch off” circuits underlying symptom expression is the goal of psychiatric and behavioral therapy. The issue with computing EEG biomarkers is that established symptom reduction strategies are generally slow compared to symptom provocation. Here, we survey the different approaches for creating such a contrast between “high” and “low” symptom expression from which it may be possible to extract a reliable, individualized EEG-derived biomarker of brain circuit activity. We will summarize symptom provocation methods and discuss possible strategies for achieving fast symptom reduction and switching between both states.

## 2. Symptom provocation for biomarker identification

The rapid switching between “on” and “off” states is required to identify biomarkers of brain circuit activity, it is clear that not all psychiatric disorders are equally amenable to this approach. Instead of evaluating a specific psychiatric diagnosis, a more promising approach may be considering trans-diagnostic symptoms experienced across different psychiatric disorders. Considering the existing literature, it seems that symptoms such as anxiety (Holt and Andrews, 1989), phobic fear (Schienle et al., 2007), obsessive thinking and compulsive avoiding behavior (Maia et al., 2022) and craving (Milivojevic et al., 2020) (either for a substance or for food) may be more easily provoked and resolved more rapidly than other symptoms, such as worry, dysphoria/dysthymia and mania/hypomania. We will discuss insights from psychopharmacological interventions for symptom provocation and then focus our discussion on selected symptoms which we consider promising candidates for behavioral interventions for both symptom provocation and symptom reduction and for which physiological models exist that relate the severity of symptom expression to the degree of activity of a specific dysfunctional brain circuit.

### 2.1. Psychopharmacological interventions for symptoms provocation

There is extensive research on how EEG and TMS-EEG are affected by the neurophysiological changes induced by pharmacological interventions (Ziemann, 2004). However, pharmacological interventions have a long history in psychiatric symptom provocation to yield insights into the biological basis of different disorders. For example, a recent study used isoproterenol to evoke physiological and emotional symptoms of anxiety/fear in patients with anorexia nervosa (Khalsa et al., 2015). When psychopharmacological agents are applied to provoke psychiatric symptoms, bottom-up and top-down approaches are used (D’Souza et al., 1999). An example of the bottom-up approach is the study of MHPG (methoxy-hydroxy-phenylethylene glycol) accumulation and anxiety in response to yohimbine administration. Studies in animal models suggest that α_2_ adrenergic antagonists stimulate the brain stem noradrenergic nucleus, the locus coeruleus, increase norepinephrine release and its conversion to MHPG, and produce anxiety-related behaviors (D’Souza et al., 1999). Conversely, in the top-down approach, a psychopharmacological agent is applied to produce a distinctive behavioral response without the underlying neurophysiological mechanism of action being completely understood, e.g., in the induction of panic attacks with sodium lactate (Margraf et al., 1986). The bottom-up approach tests a hypothesis derived from pre-clinical studies, and a top-down approach generates clinically-based hypotheses tested in the laboratory.

Historical examples of the application of psychopharmacological agents for research in psychiatric disorders are the works of Carlsson et al. (1957), Carlsson and Lindqvist (1963), Andén et al. (1970), and Nybäck and Sedvall (1970), which demonstrated the role of dopamine in the pathophysiology of schizophrenia. It was shown that provocation with amphetamines induced stereotypic behaviors in humans (Rylander, 1969), mediated by the dopaminergic system (Randrup, 1970). In subsequent symptom provocation studies, amphetamines enhanced mesolimbic dopamine activity associated with positive symptoms in schizophrenia and reduced mesocortical dopamine activity associated with negative symptoms (Davis et al., 1991). Provocation with psychostimulants in combination with neuroreceptor imaging suggested that patients with schizophrenia respond to provocation with amphetamine with elevated dopamine release compared to controls, highly correlated with an increase in psychotic symptoms (Abi-Dargham et al., 1998). In this case, switching on was achieved with psychostimulants. Switching off is the aim of treatments for positive symptoms in schizophrenia, whether pharmacologically (e.g., primarily DA-D2 antagonists or serotonin and adrenaline receptors targeted by antipsychotic drugs) or through psychotherapy (Keepers et al., 2020). However, neither pharmacotherapy nor psychotherapy rapidly reduce symptoms (on the order of minutes). Furthermore, repeated pharmacological switching (as is possible, for example, for the depth of anesthesia by adjusting the flow rate of intravenous sedatives) seems less applicable for psychiatric symptoms. For example Lorazepam which is often prescribed for short-term relief of anxiety symptoms takes 1–3 min if administered intravenously, but has an elimination half-life of ca. 14 h (Ghiasi et al., 2023) and is therefore not suitable for repeated pharmacological switching. The different concentrations of the medication also have different physiological effects on the EEG and TMS-EEG measures that would be difficult to disentangle from the influence of symptom severity. Behavioral approaches are likely more promising for extracting biomarkers of different endogenous brain states during a relatively short measurement.

### 2.2. Behavioral interventions for symptom provocation

Symptom provocation by environmental triggers is experienced daily by many psychiatric patients. However, transferring this to a laboratory setting in a reliable and controlled way is not trivial.

#### 2.2.1. Symptom provocation of trauma-related symptoms

One example of behavioral interventions being applied for research purposes is PTSD. In order to provoke symptoms in PTSD, tasks of passive emotion processing are employed. Examples include emotional faces that convey threat [e.g., angry, fearful (Shin et al., 2005)], aversive imagery (e.g., mutilated bodies, images of violence) (Lanius et al., 2007), trauma-specific cues (such as words, noises (Bremner et al., 1999b), pictures), and autobiographical scripts (Bremner et al., 1999a) - the patient’s narratives about the traumatic experience that are read to the patient in order to provoke symptoms of PTSD. This script-driven imagery paradigm is based on earlier psychophysiological studies by Lang (1979) and Pitman et al. (1987). Lang demonstrated a significant increase in heart rate and skin conductance measures in phobic subjects during imagery of their phobic objects or situations. Pitman used five individualized scripts portraying actual experiences from the subject’s past as well as six standard scripts portraying various hypothetical experiences (two neutral, a combat experience, a positive experience, an action experience and a fear experience) to demonstrate exaggerated physiologic arousal during recollection of traumatic experiences in PTSD.

#### 2.2.2. Symptom provocation in major depressive disorder

There is a significant need to achieve a better understanding of the neurophysiological basis of major depressive disorder (MDD), and to develop new and more effective therapies, including non-pharmacological therapies. Fluctuations in mood in most cases occur relatively slowly and achieving the rapid switching between high and low degrees of dysphoria that is needed for the approach presented here is likely difficult. Nevertheless, various methods of symptom provocation in MDD have been used successfully to advance this research (Martin, 1990). We summarize some of promising approaches below, which could be adapted to the proposed rapid psychiatric symptom switching framework.

One approach that has been used successfully is the use of a script driven depressive vs. happy autobiographical memory task. This has been used in combination with positron emission tomography to investigate neural pathways mediating transient mood changes in unipolar depression (Liotti et al., 2002). However, in another study it was shown that autonomic markers of symptom severity did not return to baseline after emotional provocations using an autobiographical memory task (Lin et al., 2022). Other studies have used a random number generation task (Shinba, 2014), speech writing and delivery task (Cyranowski et al., 2011), mirror tracing task (Rottenberg et al., 2007) or a video with sad emotional content (Rottenberg et al., 2003). A mood provocation task (combining elements of music associated with sad mood and autobiographical recall presented on a CD player) was also used to investigate the vulnerability of remitted depressed patients to the (re)activation of depressive thinking styles triggered by temporary dysphoric states (Segal et al., 2006). Finally, with regard to behavioral interventions, an emotional non-musical and musical stimuli paradigm was used to investigate the neural processing of emotionally provocative auditory stimuli in MDD (Nummenmaa et al., 2014; Lepping et al., 2016).

In terms of pharmacological intervention to provoke symptoms of MDD, the administration of alpha interferon (IFNa) has been found to cause flu-like symptoms as well as depressive symptoms, including depressed mood, dysphoria, anhedonia, helplessness, mild to severe fatigue, anorexia, and weight loss, hypersomnia, psychomotor retardation, decreased concentration, and confusion (Yirmiya et al., 1999). However, this is likely an unspecific modulation and the pharmacological intervention is difficult to reverse rapidly.

#### 2.2.3. Symptom provocation of specific anxiety

Anxiety is either seen as a symptom of different psychiatric disorders (e.g., generalized anxiety disorder, social anxiety disorder, PTSD, borderline personality disorder) or secondary to other symptoms experienced within a psychiatric disorder–within schizophrenia or caused by intrusive thoughts in OCD or PTSD, for example. Provoking anxiety is easier the more specific the fear is–speaking in front of an audience or solving a difficult task in front of the experimenter in social anxiety disorder, for example. Video-based symptom provocation can also induce anxiety in laboratory settings (Miskovic and Schmidt, 2012; Boehme et al., 2014). In PTSD, anxiety-provoking pictures or sounds containing violence or combat (depending on what caused the trauma) can directly induce anxiety, trigger memories that are anxiety provoking, or a combination of the two. In contrast, worry is a more generalized symptom, and a one size fits all approach will likely not be successful as the cognitive content may differ from person to person. In this case, an individually tailored approach might be more promising.

#### 2.2.4. Symptom provocation of intrusive thoughts

Intrusive thoughts are often provoked by environmental stimuli and are experienced in OCD, PTSD, major depressive disorder, and bipolar disorder. Here we will focus on intrusive thoughts experienced in OCD. Three main conceptualizations of OCD have been described depending on which faculty is taken as central: affective (anxiety/distress), volitional (compulsive), or cognitive (obsessional) (Denys, 2011). In the past, OCD was classified as an anxiety disorder due to the relevance of anxiety in the clinical presentation. However, OCD and the OCD spectrum disorders are grouped under a distinct category in the DSM-5 to take into account that increased anxiety can lead to fearful intrusive obsessive thoughts and volitional (compulsive) behavior but that anxiety can also be secondary to obsessional thoughts and the volitional attempts to regain cognitive control–[it has, however, also been hypothesized that the compulsive behavior itself could be the primary driver of symptoms of OCD (Robbins et al., 2012)]. Subsequently, we will also discuss intrusive thoughts (different from rumination) in the context of OCD, but the approaches also apply to intrusive thoughts as a symptom of other psychiatric disorders.

The state of neural activation in the target region seems important for the efficacy of TMS (Silvanto et al., 2008)–the current TMS treatment protocol for the treatment of OCD that is approved by the U.S. Food and Drug Administration applies symptom provocation before each stimulation session to elicit a moderate level of obsessional distress reported by patients (Food and Drug Administration, 2020). The underlying (although untested) assumption for the application of symptom provocation before treatment is that symptom provocation induces the reconsolidation of fear and distressing memories into long-term memories, which can be disrupted by neural stimulation during this susceptible period (Forcato et al., 2007; Agren et al., 2012; Schiller et al., 2013). Symptom provocation is believed to activate the cortico-striato-thalamo-cortical circuitry, mainly in the right hemisphere, which can be targeted by TMS (Saxena et al., 2001). Stimulating circuits that are functionally activated by symptoms is thought to increase the efficacy and specificity of TMS-induced plasticity as it allows for specific modulation of neural populations most affected by symptoms and stress (Saxena et al., 2001; Silvanto et al., 2008). Notably, whereas symptom provocation, typically as a mechanism for exposure therapy, is an established part of psychotherapy in the treatment of a number of psychiatric conditions, the additional benefit of current therapeutic TMS protocols for OCD is only moderate vs. symptom provocation alone (in the placebo condition, i.e., with sham TMS) (Carmi et al., 2019). In the case of PTSD, recent trials even indicate that TMS can reduce the therapeutic benefit of symptom provocation alone (Isserles et al., 2021). The present challenges of achieving an effective interaction between symptom provocation and therapeutic neuromodulation is part of the motivation for the development of new personalized and biomarker-based brain stimulation protocols.

An individual-tailored symptom provocation hierarchy is designed with the patient prior to treatment with TMS, such as the seven-step process following a provocation hierarchy design proposed by Maia et al. (2022). As described by Tendler et al. (2019), symptom provocation should be administered using an internal and external hierarchy provocation list to formulate questions that instill obsessive distress until the desired level of self-reported distress is achieved (Maia et al., 2022). The desired level of subjective self-reported distress is defined according to clinical experience with good acceptability and treatment efficacy in clinical trials (Carmi et al., 2019). Symptom provocation aims to achieve a moderate self-reported level of obsessional distress (i.e., “4–7” on a “0–10” Visual Analog Scale) before each stimulation session (Maia et al., 2022). In addition to provocation hierarchy approaches, virtual reality is also emerging as an option to provoke symptoms of OCD (Dehghan et al., 2021). There is evidence that the degree of distress during provocations done immediately prior to TMS for OCD is related to symptom improvement more so than the variability of distress, between-session habituation, and advancing up the hierarchy of symptom provocation hierarchy (Guzick et al., 2022). Challenges in this method include the possibility that patients may engage in compulsions (physical or, more likely, mental compulsions) during the symptom provocation, during TMS stimulation, or after TMS stimulation. These could interfere with the intensity of the symptom provocation, with the efficacy of TMS, or with the overall efficacy of the treatment, respectively.

#### 2.2.5. Symptom provocation of craving

Finally, craving is another symptom that can be provoked in a clinical setting in a relatively straightforward way. Craving is a symptom that occurs in the context of addictions (e.g., to drugs, nicotine, or alcohol) and eating disorders (Mussell et al., 1996; Greeno et al., 2000; Waters et al., 2001; Jarosz et al., 2007; Ng and Davis, 2013), including bulimia nervosa and binge eating disorder. Craving can be cue-induced (for example, presenting a particular smell or picture). It has also been proposed that there are some similarities between OCD and aspects of craving (Modell et al., 1992). In a laboratory setup, administering a cue reactivity task using pictorial pictures of either food or the drug is a possible way to provoke craving. In summary, symptom provocation is a well-established approach for a number of symptoms and psychiatric disorders. However, approaches for rapidly reducing symptoms in an experimental setting are much less studied.

## 3. Strategies for fast-acting symptom reduction

As discussed above, pharmacological strategies, although a cornerstone of therapy in psychiatry, can only be utilized in exceptional circumstances for the purpose of the approach put forward here. Behavioral therapeutic strategies are likely to be more suitable and psychotherapy is an effective approach to symptom reduction by achieving a behavioral and/or cognitive change. The underlying explanation for different treatment approaches is the widely-used emotion regulation theory by Gross (2015). This theory assumes that emotions are reactions to the world, so to feel differently or experience a reduction of symptoms, one has to try to think or pay attention differently or act differently. However, most established techniques are designed to achieve a lasting change over weeks or months, not minutes. Conversely, methods of achieving rapid symptom reduction are generally only short-lasting and often not helpful (and sometimes harmful), such as eating to reduce cravings. Below, we discuss general approaches that may be suitable for the reduction of the symptoms proposed above, which may occur in different psychiatric disorders, including relaxation techniques, thought suppression, guided attentional distraction, focused distraction, as well as acceptance and the performance of the specific compulsions in a controlled manner.

### 3.1. Symptom reduction by trigger removal in anxiety disorders

Anxiety is a prevalent symptom in a number of psychiatric disorders as both a primary or secondary symptom. The more specific the anxiety is, the easier it can be modulated by provocation and reduction strategies. One example is specific phobia: When a patient with spider phobia is engaged in a behavioral approach test (BAT) involving a live spider, increased avoidance and subjective distress can be monitored. When the spider is out of sight, subjective distress and avoidance are relatively rapidly reduced. When the spider is presented again, avoidance and subjective distress rise. In this scenario, a quick switch between different states (anxious versus not anxious) can be achieved simply by presenting and removing the trigger. A more general example of this principle is the threat of predictable or unpredictable shock (Davis et al., 2010). Often combined in a single (N)eutral, (P)redictable, and (U)npredictable threat task (Schmitz and Grillon, 2012). Shock threat leads to a rapid increase arousal, which can be measured both physiologically (Hur et al., 2020) and via self-report (Balderston et al., 2017b). When the threat is predictable, this elevated arousal is transient (on the order of seconds) and only present when the cue for the aversive shock is present (Åsli et al., 2009). In contrast, when the threat is unpredictable, the elevated arousal can be sustained (on the order of minutes or longer) and persists during the entire period of elevated threat (Ameli et al., 2001). Typically, elevated arousal is probed via random presentations of a loud white noise, which elicits an acoustic startle reflex (Grillon et al., 1991). This reflex is potentiated during elevated threat (Balderston et al., 2017a).

In social anxiety, symptoms can be reduced by removing the patient from the situation that causes anxiety or stopping the video or other stimulus used for symptom provocation. For investigating specific phobias, removing the anxiety-provoking trigger could be a suitable method to switch behavioral states. Removal of the situation is not a therapeutic approach but a potential experimental behavioral intervention for switching on and off the respective brain circuit to enable the computation of EEG biomarkers. Moreover, it may have ecological validity since avoidance is a common behavioral response to confrontation with anxiety-provoking stimuli naturalistically.

### 3.2. Symptom reduction by relaxation, suppression, distraction, and acceptance in intrusive thoughts

Here, we consider methods for the fast-acting reduction of intrusive thoughts such as experienced in OCD. Clinical experience suggests large inter-individual differences regarding the strategies that are likely to be effective. Therefore, developing a practical approach that can rapidly reduce the frequency and intensity of intrusive thoughts in a laboratory setting depends on the individual and the nature of the intrusive thought being targeted. Note that these approaches are designed to develop an EEG-derived biomarker in a laboratory setting. They are not necessarily all suitable in a subsequent therapeutic setting that consists of a combination of symptom provocation with biomarker-guided personalized TMS.

For intrusive thoughts that induce non-specific anxiety, muscle relaxation and diaphragmatic breathing are symptomatic approaches that aim to reduce the physiological symptoms associated with the intrusive thought, which may indirectly reduce the frequency and intensity of intrusive thoughts experienced. The researcher or clinician needs to be familiar with these techniques to effectively guide the patient toward a reduced level of anxiety. Suppression is a more direct mental control technique that can rapidly and effectively manage unwanted thoughts, with several standard thought suppression paradigms available often using various different distractors. As a therapeutic strategy, thought suppression, while it could be effective temporarily (Roemer and Borkovec, 1994), may also exacerbate an existing obsessional state (Wegner, 1994; Grisham and Williams, 2009), and might become a mental compulsion. Nevertheless, thought suppression could be considered a viable approach for symptom modulation in a laboratory setting with the limitation that this approach becomes ineffective quickly and that a potential rebound effect might not coincide with the desired timing of the symptom-provocation phase.

A further approach that is also widely used therapeutically is distraction. A patient’s ability to distract themselves from intrusive thoughts, behaviors and accompanying elevated anxiety is essential when compulsions cannot be performed. The extent to which patients can distract themselves varies significantly. Based on Simon et al. (2014), attentional distraction also appears to be effective in OCD patients and thereby distinguishes OCD from other anxiety disorders. It was found that patients with OCD use self-generated distraction less frequently than controls (Amir et al., 1997). As this technique was shown to be effective for reducing clinically relevant intrusive thoughts in the short term (Najmi et al., 2009) (even though not been tested in the long term), guided attentional distraction appears to be a promising candidate approach to switch to a low-symptom state in OCD patients, using, for example, a distracting bar orientation task as in Simon et al. (2014). A similar approach to guided attentional distraction is focused distraction. Instead of performing a task as mentioned above, attention is focused on a specific different thought (Wegner et al., 1987) as opposed to the unfocused distraction strategy employed in thought suppression. One way to apply focused distraction is to ask patients to focus on the thought of a specific weekend with friends they have either enjoyed or hope to enjoy and focus on the details (Najmi et al., 2009).

A further option to achieve a reduction of intrusive thoughts is acceptance. Acceptance is based on increasing the individual’s ability to experience distressing thoughts without attempting to alter their content or frequency (Hayes et al., 1999), i.e., without trying to get rid of the thought or accompanying emotions. This strategy aims to reduce reactivity to unwanted thoughts without trying to reduce their frequency (Bach and Hayes, 2002). The underlying idea is that acceptance encourages passive observation of the unwanted thought and discourages the patient from struggling with it. Ultimately, distress typically reduces after practicing this consistently, although that is taught to not be the patient’s immediate goal. However, acceptance in most cases is not a rapid strategy compared to distraction that can work instantaneously in most circumstances.

### 3.3. Symptom reduction by acting on compulsions in OCD

Finally, patients with OCD usually try to cope with anxiety and distress caused by intrusive thoughts, impulses or images by developing repetitive acts or specific rituals. According to the DSM-5, compulsions are defined as repetitive behaviors or mental acts (e.g., praying, counting, and repeating words silently) that the person feels driven to perform. These mental acts and behaviors aim to prevent or reduce distress or prevent some event or situation, but they are either excessive or disconnected with what they are intended to prevent. However, performing the individual ritual might be an effective way to achieve a rapid, short-term reduction in symptom severity, which could be quite suitable for the purpose developed here. This approach may work in a specific subset of anxieties and compulsions. Contamination anxiety and associated hand-washing compulsions are suitable examples for the laboratory setting. Many compulsions will be difficult to perform in the environment of a TMS and EEG recording, either because they only occur in a specific environment (checking doors and locks at home, for example) or because of limitations due to the nature of the intrusive thought and the associated compulsion, such as intrusive thoughts of harming or having harmed others and seeking reassurance.

For most patients, the reassurance may last for some time, and it is difficult to provoke symptoms repeatedly. However, compulsions are not always consistently effective at reducing distress and, in some cases, can cause distress. A further source of reduced reliability is that patients sometimes must repeat compulsions because they were ineffective the first time. It is also important to consider that asking a patient with OCD to act on his or her compulsions might be effective for the proposed EEG-biomarker estimation measurement, but this is not a beneficial therapeutic approach in the long term and can even aggravate the underlying anxiety (Wittgenstein, 1975; de Haan et al., 2013). An alternative experimental symptom provocation model to induce and reduce distress in a relatively controlled manner consists of distress being triggered, e.g., with photographs relevant to their OCD symptom subtype(s), followed by removal of the photographs when the participant pushes a button (Banca et al., 2015). The participant engages in silent counting as a distraction technique between symptom provocations to help reduce distress. This technique mimics the individual symptom-reducing compulsive avoidance behaviors, but does not cause movement or muscle artifacts in the EEG recording and may lead to more homogenous group data, than the use of individual strategies.

### 3.4. Symptom reduction by consumption in craving

Craving is a prominent symptom of addiction (to drugs, nicotine, or alcohol) or eating disorders. As discussed earlier, craving can be induced relatively easily using a suitable cue, but unlike anxiety (see Section “3.1. Symptom reduction by trigger removal in anxiety disorders” above), cue removal does not by itself reduce craving. There are similarities between OCD and some aspects of craving (Modell et al., 1992), as both are associated with intrusive thoughts that cause significant distress and, in the case of OCD, cause significant anxiety or cause the patient to act on certain compulsions in order to control them or in case of addiction lead to consumption of a substance. Similarly to acting on specific compulsions of OCD, a direct, rapidly acting and controllable approach to reduce craving symptoms could be simply allowing consumption of the substance (or food) craved. Similar limitations apply as in the previous section, and this is not a therapeutically beneficial approach and may worsen the underlying addiction.

### 3.5. Symptom reduction by mindfulness training

Another approach that has been discussed in the context of addiction is mindfulness training. Mindfulness involves two primary elements: focused attention and open monitoring (Lutz et al., 2008). During focused attention, attention is concentrated on a sensory object (often the sensation of breathing, but interoceptive and proprioceptive body sensations or external visual foci can also be used) while one acknowledges and then disengages from distracting thoughts and emotions. Focused attention practices often precede the practice of open monitoring, in which one observes both the arising of mental contents and the field of awareness in which those contents arise (Garland and Howard, 2018). These techniques have proven beneficial in several different clinical trials in reducing cravings and could be a promising approach to achieve a reduction of cravings in a laboratory setup where the goal is to switch between different brain states within minutes. A downside of this approach may be that it requires a significant amount of prior training and not all study participants are likely able to perform this strategy effectively (Witkiewitz and Bowen, 2010; Garland et al., 2017; Spears et al., 2017).

## 4. Discussion

### 4.1. Summary

In this paper, we have considered different approaches that may be applicable to achieve a rapid switching between states of low and high symptom severity in a laboratory setting to extract EEG and TMS-EEG signatures of the fluctuating activity of underlying brain circuits. We have presented anxiety, intrusive thoughts, and craving as exemplary symptoms that may be suitable and relevant to different psychiatric disorders. Symptom provocation (switching “on”) is a well-established procedure in psychiatry, and it can be relatively easy to increase symptom severity in a short behavioral intervention. Conversely, rapidly reducing symptoms is generally much more difficult (switching “off”).

Strategies that are helpful therapeutically may not be suitable for the experimental paradigm motivating this study. Specifically, strategies requiring ongoing cognitive or behavioral volitional activity throughout the “low symptom severity” period would introduce an additional confounder in brain states. EEG and TMS-EEG-derived biomarkers might then be sensitive to this volitional action, as opposed to the brain circuit activity that underlies the symptom. Similarly, strategies that may be suitable for the experimental approach, where a rapidly induced short-lasting change is required, may not be advisable in certain clinical situations. Specifically, a symptom reduction in OCD may be achieved by executing specific compulsions (such as hand washing) or consuming the object of craving (such as eating food in binge eating behavior, or smoking cigarettes in tobacco use disorder) in a laboratory setting. Alternatively, removing the triggering stimuli, possibly aided by the participant having control over this avoidance-like behavior, may also be effective and perhaps more controllable.

Whereas the “threat of shock paradigm” presented above is an example for the effective experimental induction of anxiety, this paradigm cannot easily be adapted to other symptoms. The use of script driven imagery on the other hand seems to be a promising generalizable approach to realize the goal of rapid psychiatric symptom switching, when combined with intermittent recovery periods to reverse the provocation. Pitman et al. (1987) played a relaxation instruction tape prior to starting the experiment, using the approach of guided attentional distraction. Data from Lang (1979) demonstrates that imaging of relevant phobic content prompts a specific increase in the amplitude of physiological arousal. This suggests that intrusive thoughts caused by pathologic emotional networks can be instigated in the laboratory through various means, including imagery, and that the activation of these networks is reflected not only in subjective reports but also in specific patterns in the EEG and TMS-EEG response.

The desired experimental paradigm of rapid switching between two states that differ only in symptom severity, and that can be achieved during a TMS/EEG measurement in a laboratory setting, will likely not be feasible for all types of symptoms and in all patient populations. However, we hope that this approach will yield new insights in specific cases. A specific proof-of-concept personalized therapeutic brain intervention based on EEG markers of circuit activity would be an important milestone in developing future closed-loop EEG and TMS treatment approaches. We suggest anxiety, intrusive thoughts and craving as possible candidates for such an initial proof-of-concept study. Below, we discuss practical considerations regarding the design of the experimental paradigm motivating this investigation.

### 4.2. Design considerations

Although it is exciting to consider how much can be learned from the symptom provocation possibilities described above, it is important to consider the implementation of these possibilities within the context of good experimental design and rigorous electrophysiological data collection techniques. Accordingly, the following section will serve as a practical guide for how to properly conduct well-controlled EEG and TMS/EEG measurements while provoking psychiatric symptoms.

First, for the EEG-biomarker extraction, the cognitive-behavioral state between the two conditions should be similar. In an ideal scenario, the patient is in an otherwise neutral state in both conditions that enables the acquisition of TMS and EEG data without excessive signal artifacts (awake, seated in a chair, with relaxed scalp muscles, eyes open, fixating a visual target) and the behavioral intervention is short and limited to a few minutes between the conditions (see Figure 1). If it is necessary to use a mental task to achieve a change in symptom severity (such as visual stimuli, mental imagery or mental verbalization), similar tasks should be used during both conditions (e.g., observation of critical faces in one condition and observation of neutral faces in the other condition; or a less effective version of a focused distraction task, that is otherwise similar).

Second, symptom severity should be measured repeatedly during each condition to assess whether the intervention is effective. Physiological markers (skin conductance, heart rate, blink reflex, pupillometry) should be considered where available such that assessment of the effectiveness of an induced state could be corroborated by objective measurements. In the case of anxiety and craving, a visual analog scale can also be used. In the case of discrete symptoms that are either there or not, such as intrusive thoughts, the study participant can press a button to indicate when the intrusive thought occurred. This could be complemented with a visual analog scale for distress, since it is possible for one to have an intrusive thought without distress, e.g., during the symptom reduction phase of the experiment.

Third, the task should be practiced, and the measurement procedures should be demonstrated during a preparatory experimental session to reduce novelty, salience, and training effects during the recording. This could potentially also have another beneficial effect as practicing ahead of time might produce an experimentally-advantageous expectancy or priming effect, such that the person anticipates that the provocation will induce distress and the technique following it would reduce distress.

Regarding the neurophysiological measures, signal quality should be monitored during the measurement so that excessive artifacts (e.g., due to scalp muscle activity, eye blinks and eye movements) can be addressed. Obtaining high-quality concurrent TMS and EEG recordings is not trivial; recommendations for data acquisition and analysis have recently been summarized elsewhere (Hernandez-Pavon et al., 2023), and this is an active area of research. It is also not obvious to decide the target location and intensity of the TMS. It may be necessary to investigate different candidate stimulation parameters in a calibration session to determine the optimal location to probe the reactivity of the circuit under investigation at an intensity that achieves an optimal trade-off between maximizing cortical responses and minimizing TMS-related EEG artifacts.

### 4.3. Limitations

An important inherent limitation in the proposed approach is that only a small subset of symptoms and psychiatric conditions are likely suitable for the rapid, controlled modulation of symptom severity (such as a small number of specific phobias, other anxiety conditions with specific triggers, and OCD). While resulting TMS/EEG-derived biomarkers could yield relevant insights into those specific pathologies, it is currently unclear whether the findings are likely to generalize. This limitation could be further exacerbated if highly individualized provocation and reduction strategies are required, increasing the variability between participants, as the EEG measurement will be affected by the specific strategy employed to achieve the symptom modulation.

When evaluating strategies that could lead to a short-term symptom reduction but are not consistent with therapeutic approaches (such as smoking to reduce craving or execution of rituals to reduce compulsions), the potential negative short- and long-term effects on the study participant needs to be evaluated, and a clear ethical justification of any such strategies is required.

Another limitation concerns the neurophysiological measures: EEG is much more sensitive to the activity of superficially located brain regions than deep brain regions (and dipoles oriented orthogonally to the scalp surface instead of parallel). Even if an effective strategy to modulate symptom severity can be found, it may not be possible to derive reliable EEG markers for that symptom. Similar constraints affect the TMS-EEG data; TMS is thought to primarily activate neurons in the superficial parts of the cortical sulcal walls and the gyral crowns. It may be difficult to achieve the goal of using TMS to probe the circuits underlying the specific psychopathology under investigation if none of the nodes of the circuit are accessible to TMS stimulation. In these cases, indirect circuit stimulation through a connected superficial (accessible) area can be considered.

Note that the proposed approach depends on the assumption that changes in the EEG reflect changes in the expression of symptoms with a congruent timeline. Furthermore, because increased pathological network activity will likely coincide with involuntarily simultaneously increased activity in compensatory networks, it may be difficult to disentangle the respective EEG-signatures. Finally, even if a suitable EEG-derived biomarker can be identified, this does not automatically mean that the target circuit can be effectively modulated with a given TMS protocol.

In conclusion, it is important to match the timescale of fluctuations in circuit activity with the timescale of the neurophysiological measures. EEG data needs to be acquired over several minutes to compute reliable spectral estimates; at least dozens of TMS-EEG trials are required to extract evoked potentials and induced oscillations. For symptoms such as anxiety and craving, a stable high vs. low symptom severity condition may be achievable for tens of minutes. However, for transient symptoms, such as intrusive thoughts, symptoms may fluctuate on a much faster timescale and the underlying short-lasting physiological changes might not be accessible with the same neurophysiological measures. Lastly, different synchronization and analysis approaches may be required to identify discrete EEG events instead of slow variations.

## 5. Outlook

We believe that the current lack of reliable biomarkers that index the fluctuating activity of brain circuit activity with a temporal resolution of tens of seconds is a critical barrier in the development of a next generation of personalized therapeutic brain stimulation protocols. We hope that the approach put forward in this work may help determine such EEG-derived biomarkers and that this will lead to more effective brain intervention therapies for patients suffering from neuropsychiatric disorders.

## Data availability statement

The original contributions presented in this study are included in this article/supplementary material, further inquiries can be directed to the corresponding author.

## Author contributions

BZ, CZ, DB, and JF contributed to the conception and design of the work. BZ and JF wrote the first draft of the manuscript. CZ, NB, DB, SK, JL, RT, AT, and GZ wrote sections of the manuscript. All authors contributed to the manuscript revision and read and approved the submitted version.
